# Dereplication of Known Nucleobase and Nucleoside Compounds in Natural Product Extracts by Capillary Electrophoresis-High Resolution Mass Spectrometry

**DOI:** 10.3390/molecules20045423

**Published:** 2015-03-26

**Authors:** Junhui Chen, Qian Shi, Yanlong Wang, Zhaoyong Li, Shuai Wang

**Affiliations:** 1 Key Lab of Analytical Technology Development and Standardization of Chinese Medicines, The First Institute of Oceanography, State Oceanic Administration, Qingdao 266061, China; E-Mails: srdwjm@sina.com (Q.S.); wangyanlong92@163.com (Y.W.); lizhaoyong2056@126.com (Z.L.); wangshuai@fio.org.cn (S.W.); 2 College of Chemistry and Chemical Engineering, Ocean University of China, Qingdao 266100, China

**Keywords:** ESI-TOF/MS, dereplication, nucleobases, nucleosides, *Syngnathus acus* Linnaeus, *Hippocampusjaponicus* Kaup, *Anthopleura lanthogrammica* Berkly

## Abstract

Nucleobase and nucleoside compounds exist widely in various organisms. An often occurring problem in the discovery of new bioactive compounds from natural products is reisolation of known nucleobase and nucleoside compounds. To resolve this problem, a capillary electrophoresis-high resolution mass spectrometry (CE-HR-MS) method providing both rapid separation and accurate mass full-scan MS data was developed for the first time to screen and dereplicate known nucleobase and nucleoside compounds in crude extracts of natural products. Instrumental parameters were optimized to obtain optimum conditions for CE separation and electrospray ionization-time-of-flight mass spectrometry (ESI-TOF/MS) detection. The proposed method was verified to be precise, reproducible, and sensitive. Using this method, known nucleobase and nucleoside compounds in different marine medicinal organisms including *Syngnathus acus* Linnaeus; *Hippocampus* *japonicus* Kaup and *Anthopleura lanthogrammica* Berkly were successfully observed and identified. This work demonstrates that CE-HR-MS combined with an accurate mass database may be used as a powerful tool for dereplicating known nucleobase and nucleoside compounds in different types of natural products. Rapid dereplication of known nucleobase and nucleoside compounds allows researchers to focus on other leads with greater potential to yield new substances.

## 1. Introduction

Natural products are historically and currently the most consistently successful source of drug leads. Natural product are likely to continue to be sources of new commercially viable drug leads because the chemical novelty associated with natural products is higher than that of any other source [1]. However, the past two decades have witnessed a decrease in natural product drug discovery efforts in the pharmaceutical industry because of technological challenges in matching historical discovery paradigms with modern drug discovery strategies [2]. Most studies dealing with natural product identification following the classical approach of isolation and elucidation have encountered the bottle-neck of time-consuming procedures and repeated purification steps, which are probably the core limitations in natural product research [3]. Thus, to expedite the discovery of new leads and avoid reisolation of previously known compounds, discriminating between known *versus* new compounds as early as possible is crucial [4]. This process, which is called “dereplication” [5,6,7], enables the efficient use of human and financial resources so that efforts can focus on the discovery of structurally novel compounds [8,9,10].

In recent years, several dereplication methods for rapid identification of previously known compounds in different natural products have been reported [2,3,4,11,12,13]. Among these methods, high resolution mass spectrometry-based dereplication is critical to modern natural product dereplication pipelines, which are processes of characterizing natural products via an established infrastructure of structural analyses to identify known molecules [3,4,11,12].

Nucleosides and their bases regulate and modulate various physiological processes in the body and exhibit multiple bioactivities [14,15,16,17,18], such as antiplatelet aggregation as well as antiarrhythmic, antioxidant, antiseizure, and antitumor effects, among others. Given their benefits to human health, these compounds have recently gained increased research attention in the natural product field, and many of them have been found in several traditional Chinese medicines [18,19,20], marine organisms [21,22,23,24], and food sources [16,17,25,26]. However, since nucleobase and nucleoside compounds exist widely in various organisms, a major problem in the discovery of new biologically active compounds from natural products involves reisolation of known nucleosides and their bases [21,27,28,29,30]. Reisolation wastes time and resources, thereby distracting chemists from pursuing more promising leads. To solve this problem, dereplication strategies that screen crude extracts for the presence of known nucleobases and nucleosides before isolation efforts are initiated and necessary. Thus, developing a rapid and robust methodology to achieve this purpose would be of great value.

Several methods for quantifying nucleobases and nucleosides in biological fluids, food, and herbal materials, including high-performance liquid chromatography (HPLC) [20,26,31,32,33], ultra-performance liquid chromatography [34], hydrophilic interaction chromatography [19,35,36], liquid chromatography-mass spectrometry [16,17,18,37,38], capillary electrophoresis (CE) [22,39,40,41], and capillary electrophoresis-mass spectrometry (CE-MS) [42,43,44,45,46], have been reported. Several recent studies have confirmed that CE-MS is an ideal tool for analyzing nucleobases and nucleosides in different sample matrices [42,43,44,45,46]. CE-MS has attracted considerable attention over the last few years because of its utility in environmental analysis, food analysis, pharmaceutical analysis, and the like [47]. CE-MS is a sensitive and reliable method for easily analyzing electro-driven compounds in complex matrix samples [48,49,50]. However, reports on the application of CE-MS in dereplication of known nucleobase and nucleoside compounds in different natural products are unavailable. Moreover, in the CE-MS methods reported [42,43,44,45,46], low-resolution mass analyzers for CE-MS, such as ion-trap MS, and single quadrupole MS, present severe limitations for dereplication, leading to larger numbers of possible elemental compositions for a given ion. Accurate mass measurements of known or unknown compounds reduce the number of predicted elemental compositions, which ensures that database searches are conducted with the fewest possible candidates. High-resolution mass analyzers, such as TOF, Q-TOF, orbitrap can provide mass accuracies in the 0.5–5 ppm range, thereby yielding only one or very few possible elemental compositions for a given ion [51]. Thus, the goal of the present study is to develop a rapid and effective method for dereplicating known nucleobase and nucleoside compounds in natural products through CE coupled to a high-mass accuracy TOF mass spectrometer. As proof of concept, different marine medicinal organisms were selected for analysis; these organisms were chosen because of their value in traditional medicine.

## 2. Results and Discussion

### 2.1. Optimization of CE-ESI-TOF/MS Methodology

The first goal of the current study is to develop a CE-ESI-TOF/MS method that can rapidly screen and identify known nucleobases and nucleosides in crude extracts of different natural products. CE separation and TOF/MS detection conditions were developed and optimized using 15 typical nucleobase and nucleoside standards and an *A. lanthogrammica* Berkly sample.

Previous studies [42,43,44,45,46] report that volatile acids or salts can be used to separate nucleobases and nucleosides through CE-ESI-MS. Thus, ammonium acetate buffer was chosen for the separation experiment in this study, and separation conditions, including the concentration of ammonium acetate, pH, content of organic modifier, separation voltage and injection volume, were optimized using nucleobase and nucleoside standards.

The background electrolyte (BGE) concentration affects compound charge and migration velocity. Thus, several concentrations of ammonium acetate (20, 25, 30, 35, and 40 mmol/L) were evaluated. As the ammonium acetate concentration increased, the capillary current increased, the retention time was delayed, and the resolution of all peaks decreased. Considering the importance of balancing the separation time and resolution, 30 mmol/L ammonium acetate was finally selected as the BGE (Supplementary Information, Figure S1).

The pH of the BGE is one of the most important parameters affecting CE separation. Given the different pKa values of nucleobases and nucleosides, pH values directly influence the ionization degree and migration order of target compounds. Separation in the pH range of 9.5–10.5 was investigated. As the pH increased, a time-delay of nucleobase and nucleoside peak appearance occurred and peaks noticeably broadened. The optimum pH of the BGE was found to be 9.9 based on the analytical resolution and good peak shapes obtained (Supplementary Information, Figure S2).

Different ratios of organic modifiers (methanol) in the buffer solution, injection volumes, and separation voltages were also optimized, and relevant results for organic modifier selection are shown in Supplementary Information Figures S3. In summary, the best peak resolution was obtained under the following conditions: in an aqueous electrolyte containing 30 mmol/L ammonium acetate (with 5% methanol, pH adjusted to 9.9 by ammonium hydroxide), injection pressure of 50 mbar, injection time of 24 s, and separation voltage of 25 kV.

ESI-TOF/MS parameters were optimized to obtain the highest signal intensity. The parameters to be optimized included the sheath liquid composition and flow rate, nebulizer pressure, drying gas flow rate and temperature, capillary voltage, and fragmentation voltage. Among these parameters, the composition and flow rate of the sheath liquid can exert the greatest effects on ionization efficiency and analyte sensitivity. The sheath liquid is used to maintain the stabilities of the current and MS signals. Methanol and isopropanol are two organic reagents often used as sheath liquids. Previous studies [52,53] indicate that 50%, 75%, or 100% methanol or isopropanol (containing 1% formic acid) may be used separately as sheath fluids. The results of the present work show that 50% methanol-water solution containing 1% formic acid provides the best signal-to-noise (S/N) ratio in MS.

The formic acid content in the sheath fluid was optimized. We tested formic acid concentrations of 0.25%, 0.5%, 0.75%, and 1%, and results showed that the S/N ratio obtained from 0.25% formic acid is the best among the ratios recorded. Therefore, the final composition of the sheath liquid included 50% methanol and 50% water solution (v/v) containing 0.25% formic acid. Testing of the effects of sheath liquid flow rate ranging from 2 μL/min to 6 μL/min indicated that a satisfactory MS response for all 15 nucleobases and nucleosides may be obtained at a flow rate of 3 μL/min.

The fragmentor affects the sensitivity of the ESI-TOF/MS method, and different compounds require different fragmentors. Therefore, the fragmentation voltages of each of the 15 nucleobases and nucleosides examined in this work were optimized individually, and the best fragmentation voltages obtained were in the range of 120–150 V.

Literatures [54,55,56,57] have revealed that the frequent drops in current for CE-ESI-MS analysis can be attributed to the effect of the nebulizing gas pressure during injection and electrophoretic separation. In this study, proposed strategy by Dominguez-Alvarez *et al.* [55], called programmed nebulizing gas pressure mode, is applied during the different steps of analysis to minimize the drops in current.

**Figure 1:**
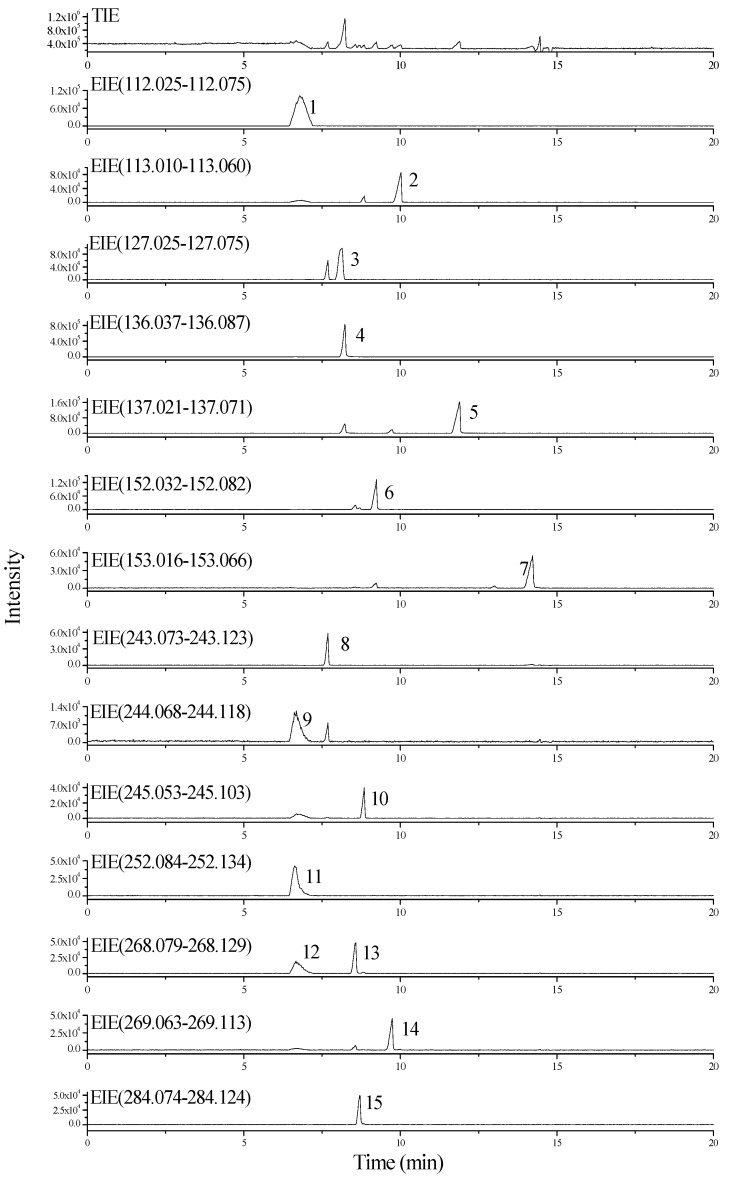
CE-ESI-TOF/MS TIE and EIEs of 15 nucleobase and nucleoside standards. Peak 1. cytosine; 2. uracil; 3. thymine; 4. adenine; 5. hypoxanthine; 6. guanine; 7. xanthine; 8. thymidine; 9. cytidine; 10. uridine; 11. cordycepin; 12. adenosine; 13. 2'-deoxyguanosine; 14. inosine; 15. guanosine. Numbers in parentheses of each panel are the molecular masses of the singly charged ions [M+H]^+^. Peak 12 (adenosine) and peak 13 (2'-deoxyguanosine) in Figure 1 have the same mass.

Under optimal CE-ESI-TOF/MS instrument conditions, typical mass electropherograms [total ion electropherograms (TIEs)] and extracted ion electropherograms (EIEs) may be obtained as shown in Figure 1. Good separation resolution was obtained from most of the nucleobases and nucleosides. Although several compounds were not completely separated, rapid identification of known nucleobases and nucleosides can be done through accurate extraction of the molecular masses of the [M+H]^+^ ion peaks obtained (Supplementary Information, Table S1).

### 2.2. Validation of the CE-TOF/MS Method

Under optimal instrument conditions, the precision of the CE-ESI-TOF/MS method was calculated using the migration times and peak areas of ions extracted from the target compounds as indices. Results show that the relative standard deviations (RSDs) of the peak areas of all 15 nucleobases and nucleosides were lower than 15.74% (Table 1) and that the RSDs of the migration times of these compounds were lower than 1.67%. These data reflect the good precision of the proposed method.

Intraday and interday variations were next used to evaluate the reproducibility of the developed method. For the intraday precision test, six replicate samples (*A. lanthogrammica* Berkly) prepared independently were analyzed within a single day. For the interday precision test, six replicate samples were examined over three consecutive days. Relevant results are shown in Table 1. The RSD values of peak areas for intraday and interday reproducibility were in the range of 6.87%–16.11% and 9.38%–24.34%, respectively, demonstrating good reproducibility of the method. Overall, the CE-ESI-TOF/MS method developed to dereplicate nucleobase and nucleoside compounds in natural products offers good precision and reproducibility.

**Table 1:** Precision, intraday and interday reproducibility, LODs and measured accurate mass error of nucleobases and nucleosides determined by CE-ESI-TOF/MS.

	Analytes	Precision RSD, %; *n* = 6	Reproducibility RSD, %; *n* = 6		LOD (µg/mL)	Error (ppm)
			Intra-Day	Inter-Day		
1	Cytosine	8.85	10.97	22.27	0.25	1.44
2	Uracil	9.87	10.14	22.63	0.25	2.18
3	Thymine	14.56	7.64	24.34	0.20	1.54
4	Adenine	9.60	8.60	22.15	0.03	0.21
5	Hypoxanthine	8.91	12.26	19.71	0.12	−0.64
6	Guanine	10.93	10.56	23.42	0.20	1.41
7	Xanthine	11.45	11.25	18.84	0.25	−1.32
8	Thymidine	15.74	11.72	23.33	0.50	1.54
9	Cytidine	14.75	ND ^a^	ND ^a^	1.00	−1.22
10	Uridine	14.85	10.57	19.00	0.75	3.21
11	Cordycepin	13.34	6.87	9.38	0.25	0.33
12	Adenosine	13.96	14.04	17.48	1.00	−1.23
13	2'-Deoxyguanosine	12.57	8.87	19.47	0.50	−1.23
14	Inosine	13.79	16.11	17.32	0.75	0.20
15	Guanosine	14.58	8.34	19.52	0.75	−0.86

^a^ Not detected.

The mass concentration at an S/N ratio of 3 was set as the detection limit of the instrument. The detection limits of all 15 nucleobases and nucleosides were within the range of 0.03–1.00 µg/mL. In fact, the detection limits of nucleobases and nucleosides determined by the present method were lower than those of obtained using traditional CE-MS methods [17]. The results fully satisfy requirements for rapid screening and dereplication of nucleobase and nucleoside compounds in real samples of natural products. Errors in mass measurement were ≤3.21 ppm in all cases, which indicates that the method enables unique assignment of the elemental compositions of the peaks. The reliability of most screening and dereplication methods depends heavily on the ruggedness of the TOF instrument to provide accurate mass measurements consistently within a fixed mass error tolerance. In typical cases, measurements of accurate masses within 5 ppm are widely accepted to verify elemental compositions [58,59].

### 2.3. Dereplication of Nucleobase and Nucleoside Compounds in Real Natural Product Samples

The foundation of the present CE-ESI-TOF/MS dereplication procedure is the construction of a database for identifying known nucleobases and nucleosides in different natural products. This study establishes an accurate relative molecular weight database containing common known nucleobase and nucleoside compounds. To rapidly screen and dereplicate these compounds in natural products, signal extraction was performed on the CE-ESI-TOF/MS TIE of each compound using accurate relative molecular mass data. TOF/MS is a high-resolution MS technique. A specific mass can be extracted in smaller mass windows from full-scan mode, and extracted ions with small windows can significantly reduce background noise, improve the S/N ratio, and reduce the occurrence of erroneous results [23,59]. In this study, EIEs using a mass window width of 50 mDa ([M+H]^+^ ± 25 mDa) was used for identification of nucleobase and nucleoside compounds. The examples shown below demonstrate the workflow of the dereplication methodology and illustrate its utility for nucleobase and nucleoside compound dereplication in natural products.

**Figure 2:**
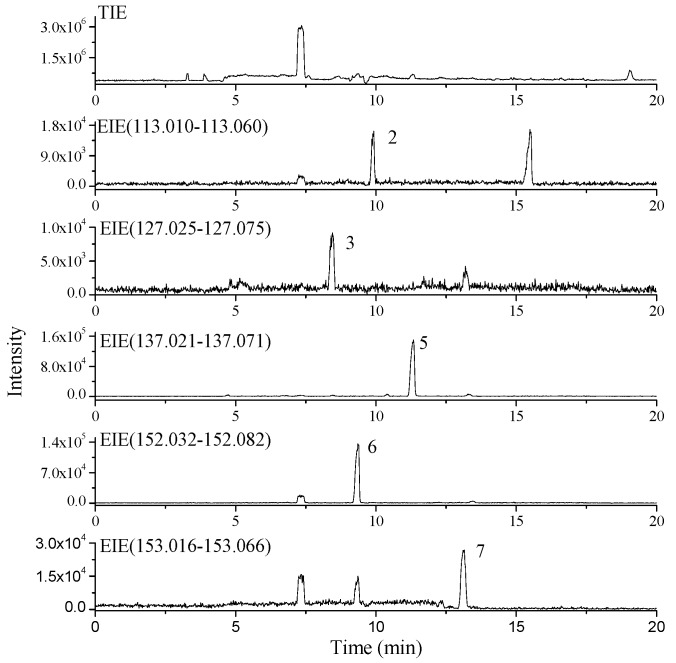
CE-ESI-TOF/MS TIE and EIEs of nucleobase compounds in the extract of *S. acus* Linnaeus. For peak assignment, see Figure 1.

**Figure 3:**
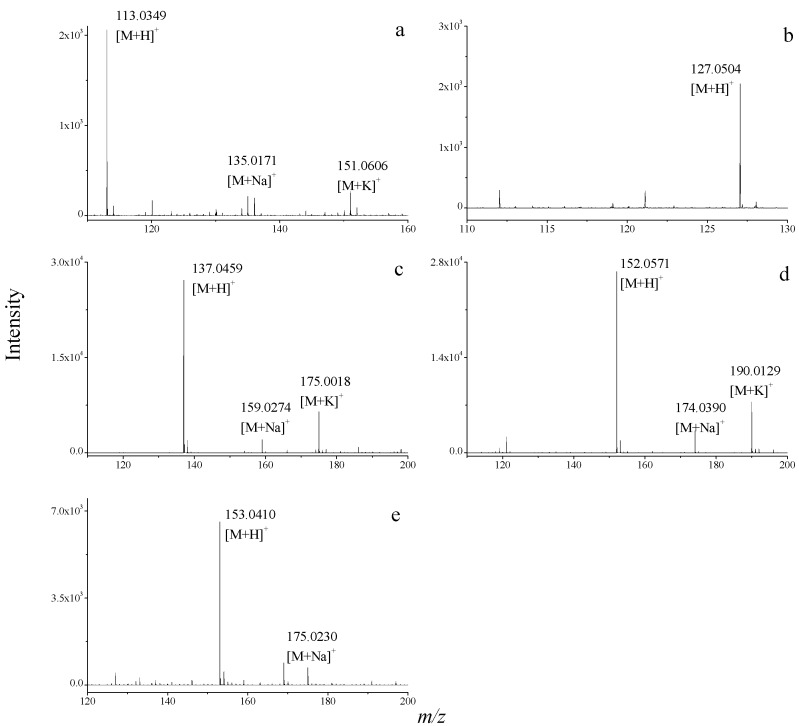
ESI-TOF/MS spectra of (**a**) uracil; (**b**) thymine; (**c**) hypoxanthine; (**d**) guanine; (**e**) xanthine in the crude extract of *S. acus* Linnaeus.

A crude extract of *S. acus* Linnaeus was analyzed using CE-ESI-TOF/MS to obtain its TIE (Figure 2) under positive ion mode. The accurate relative molecular mass data of all known nucleobase and nucleoside compounds in the database were then used to extract signals from the TIE. In Figure 2, five obvious EIE peaks were obtained through signal extraction. These five nucleobases were preliminarily identified as (a) uracil, (b) thymine, (c) hypoxanthine, (d) guanine, (e) xanthine. The ESI-TOF/MS spectra of these compounds are provided in Figure 3.

To illustrate the value of the proposed protocol, the process described below exemplifies a typical peak identification process in TOF/MS analysis. Peak 5 in Figure 2 has a migration time of 11.3 min. Peak 5 corresponds to *m*/*z* 137.0459, *m*/*z* 159.0274 and *m*/*z* 175.0018 (Figure 3c), resulting from [M+H]^+^, [M+Na]^+^ and [M+K]^+^, respectively. A possible molecular formula for this compound was thus deduced as C_5_H_4_N_4_O using Masshunter software. Isotopic peaks of the quasi-molecular ion peak were matched using Masshunter software. Here, the measured value clearly matches the calculated value. Therefore, the molecular formula of this compound was confirmed as C_5_H_4_N_4_O, and the compound was identified as hypoxanthine. The four other nucleobase compounds in the crude extract of *S. acus* Linnaeus were also detected and identified similarly.

CE-ESI-TOF/MS was used to screen and dereplicate nucleobase and nucleoside compounds in other natural product samples. Results show that *H. japonicus* Kaup contains hypoxanthine, guanine, xanthine, uracil, and thymine, while *A. lanthogrammica* Berkly contains adenine, hypoxanthine, xanthine, guanine, cytosine, thymine, uracil, uridine, adenosine, guanosine, thymidine, inosine, 2'-deoxyguanosine, and cordycepin. According to the results described, CE-ESI-TOF/MS combined with accurate mass screening can meet requirements for the rapid screening and dereplication of known nucleobase and nucleoside compounds in different natural products. Rapid dereplication of these compounds allows researchers to focus on other leads with greater potential to yield new substances. According to literature [3,4,11,12], LC-HR-MS-based dereplication is popular to modern natural product dereplication pipelines. However, CE-TOF/MS as a suitable alternative to LC-TOF/MS [11,12] for dereplication has its own distinguishing features and advantages. CE is becoming increasingly recognized as an important separation technique, due to its high resolution, small sample volumes, extraordinary small solvents consumption and rapid separation with high efficiency.

## 3. Experimental Section

### 3.1. Reagents, Materials and Standard Solutions

HPLC-grade methanol, acetone, and acetonitrile were obtained from Merck (Darmstadt, Germany). Analytical-grade sodium hydroxide and ammonium acetate were provided by Sigma-Aldrich (St. Louis, MO, USA). Water was prepared using a Millipore Milli-Q water purification system (Bedford, MA, USA). *Anthopleura lanthogrammica* Berkly samples were obtained from a fisherman in Qingdao, China, and *Hippocampus* *japonicus* Kaup and *Syngnathus acus* Linnaeus samples were purchased from a drugstore in Qingdao of China. All of the samples were dried, comminuted using a pestle and mortar, and then sieved through a 0.4 mm stainless steel sieve before use.

Standards of adenine, hypoxanthine, xanthine, guanine, cytosine, thymine, uracil, cytidine, uridine, adenosine, guanosine, thymidine, inosine, 2'-deoxyguanosine, and cordycepin were purchased from Sigma-Aldrich. A stock solution of each standard (0.1 mg/mL) except cordycepin was prepared in 5% aqueous ammonia solution. Cordycepin (0.1 mg/mL) was dissolved in 10% aqueous methanol. These solutions were stored under refrigeration (4 °C). Mixed standard solutions were prepared by diluting stock standard solutions to the desired concentrations; here, the concentration of each diluted standard was 40 μg/mL. Both the buffer and standard solutions were filtered through a 0.45 μm nylon membrane filter and degassed by ultrasonication for approximately 10 min before use.

### 3.2. Sample Preparation

Sample extraction was performed as described previously [22]. Briefly, dried sample powders (0.2 g) and 20 mL of 60% (v/v) aqueous methanol were placed in a flask that was then placed in an ultrasonic bath for 15 min. Extraction was repeated two more times, and all extracts were combined. The combined extracts were filtered and evaporated to dryness using a rotary evaporator at 55 °C. Then, the residue was dissolved in 10 mL of a mixed solvent of 2% (v/v) ammonia and 4% (v/v) methanol. After centrifugation at 6000× *g* for 10 min, the solution was filtered through a 0.45 μm nylon filter membrane and degassed before use.

### 3.3. CE-ESI-TOF/MS Conditions

Analyses were carried out in an HP^3D^ CE system (Agilent Technologies, Palo Alto, CA, USA) coupled to a Model G1969A time-of-flight mass spectrometer (Agilent Technologies) for high-resolution MS detection through an orthogonal electrospray interface (ESI). Data were processed using Masshunter software (A02.02) in Agilent TOF/MS. Separation was carried out in a 80.5 cm × 50 μm i.d. uncoated fused silica capillary from Agilent (Waldbronn, Germany). Before first use, the capillary was conditioned with 1 M sodium hydroxide by flushing for 30 min, followed by flushing for 20 min with water and 10 min with 30 mM ammonium acetate (pH 9.9) buffer. After each run, the capillary was conditioned with Milli-Q water (5 min) and separation buffer (5 min). A solution of 30 mM ammonium acetate (pH = 9.9 adjusted by ammonia) was used as the BGE. Injections were made at the anodic end (inlet) using a pressure of 50 mbar for 24 s. Electrophoretic separation was achieved with a voltage of 25 kV and at room temperature (25 °C).

To couple the CE system to the TOF mass spectrometer, a coaxial sheath-liquid sprayer (Agilent Technologies) was used. An Agilent 1100 LC pump coupled to an accurate splitter (1:100; LC Packing, Dionex Company, Sunnyvale, CA, USA) was utilized to deliver the sheath liquid solution at a flow of 3 μL/min. The coaxial sheath liquid consisted of a mixture of methanol/water (50:50 v/v) containing 0.25% formic acid. The solution was degassed for 5 min in an ultrasonic bath before use. The TOF mass spectrometer conditions were optimized for nucleobases and nucleosides detection as follows: in positive mode; drying gas temperature, 200 °C; drying gas flow, 10.0 L/min; nebulizer pressure, 8 psi; fragmentor, 150 V; capillary voltage, 4000 V; skimmer, 65 V; OCT 1 RF Vpp, 250 V. The mass spectrometer performed scans from *m*/*z* 110 to 500 in full scan mode. Calibration reference solution obtained from Agilent was used to calibrate the mass spectrometer everyday. This TOF/MS instrument features a high mass resolving power of over 10,000 at FWHM. Thus, the deviation in a measured mass can be controlled to within 5 ppm in terms of *m*/Δ*m*. Internal calibrant was used to get the mass accuracy <5 ppm.

## 4. Conclusions

In this study, a new dereplication method was successfully established for the rapid screening and identification of known nucleobase and nucleoside compounds in natural products by CE-HR-MS combined with accurate mass database for the first time. The proposed instrumental method was verified to be precise, reproducible, and sensitive. As a proof of concept, the crude extracts from three marine medicinal organisms including *Syngnathus acus* Linnaeus; *Hippocampus* *japonicus* Kaup and *Anthopleura lanthogrammica* Berkly were used for the dereplication of known nucleobases and nucleosides. Based on the high resolution and the accurate mass measurement in full-scan acquisition, known nucleobase and nucleoside compounds (including hypoxanthine, guanine, xanthine, uracil, thymine, *etc.*) were successfully observed and identified rapidly. Overall, this new method enables the fast identification and dereplication of known nucleobase and nucleoside compounds in different types of natural products such as medicinal plants, medicinal animals, agricultural products, food materials, and microbes, among others. The identification of known nucleobase and nucleoside compounds early in the discovery process could absolve multiple laborious isolation steps, and will allow researchers to focus on other bioactive compounds with greater potential to yield new substances.

## Supplementary Materials

Supplementary materials can be accessed at: http://www.mdpi.com/1420-3049/20/04/5423/s1.
