# Supplementary material for: Dereplication of Known Nucleobase and Nucleoside Compounds in Natural Product Extracts by Capillary Electrophoresis-High Resolution Mass Spectrometry
Source: Molecules. 2015 Mar 26;20(4):5423–37. doi: 10.3390/molecules20045423 (PMC6272742; doi:10.3390/molecules20045423)
Supplement: Supplementary file 1 [file molecules-20-05423-s001.pdf]

Supplementary Materials

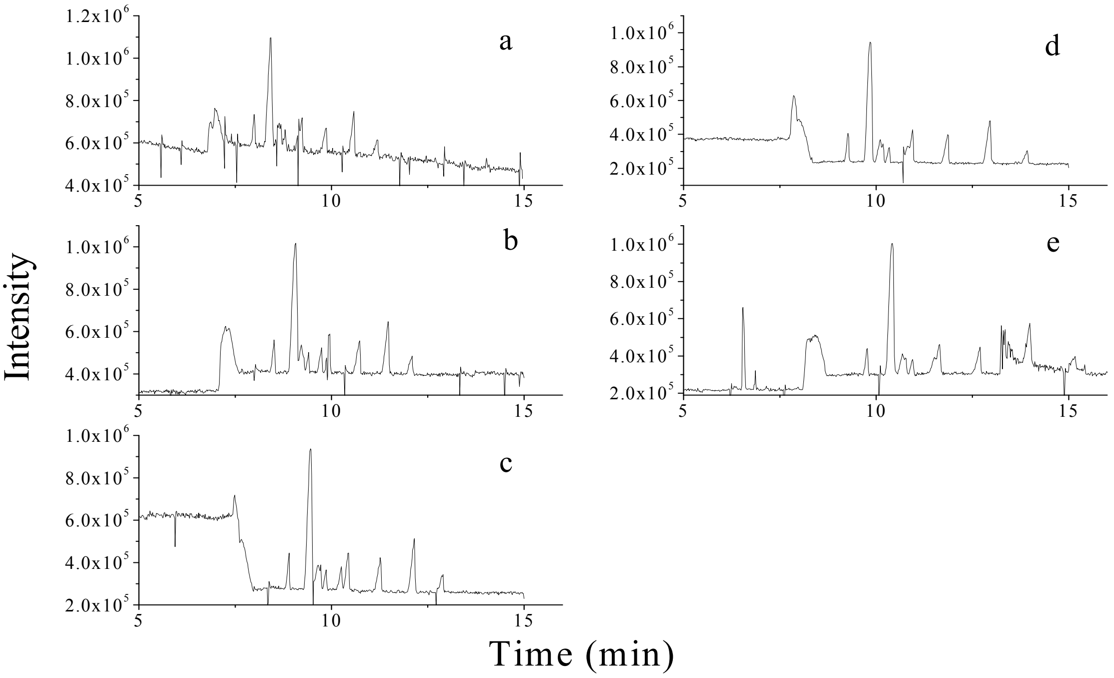

Figure S1. TIEs of mixed standards (including cytosine; uracil; thymine; adenine; hypoxanthine; guanine; xanthine; thymidine; cytidine; uridine; cordycepin; adenosine; 2'-deoxyguanosine; inosine and guanosine.) with different ammonium acetate concentrations. (**a**) 20 mM, (**b**) 25 mM, (**c**) 30 mM, (**d**) 35 mM, (**e**) 40 mM. The concentration of each diluted standard was 40 µg/mL.

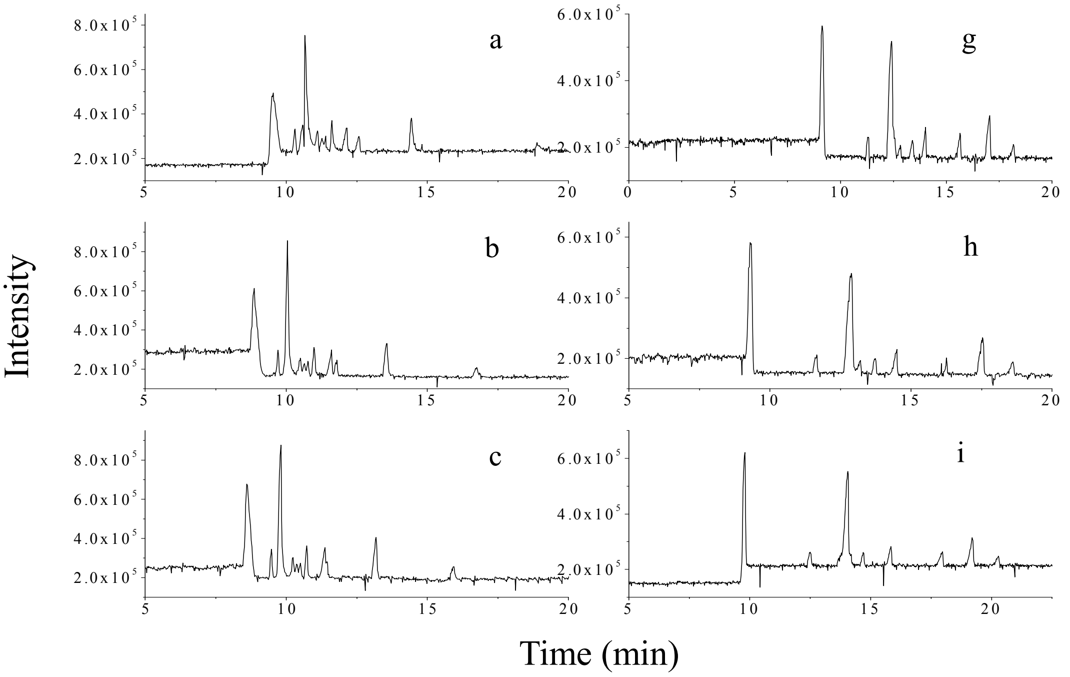

Figure S2. *Cont.*

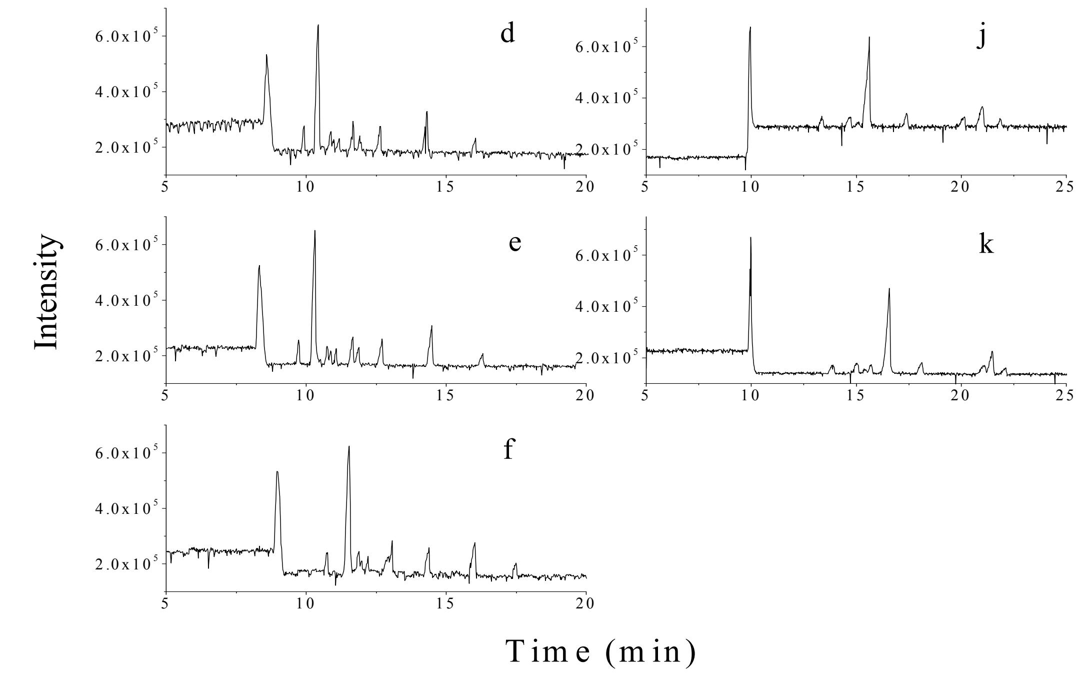

Figure S2. TIEs of mixed standards at different pH values. (**a**) pH9.5, (**b**) pH9.6, (**c**) pH 9.7, (**d**) pH 9.8, (**e**) pH 9.9, (**f**) pH 10.0, (**g**) pH 10.1, (**h**) pH 10.2, (**i**) pH10.3, (**j**) pH10.4, (**k**) pH10.5. The concentration of each diluted standard was 40 μg/mL.

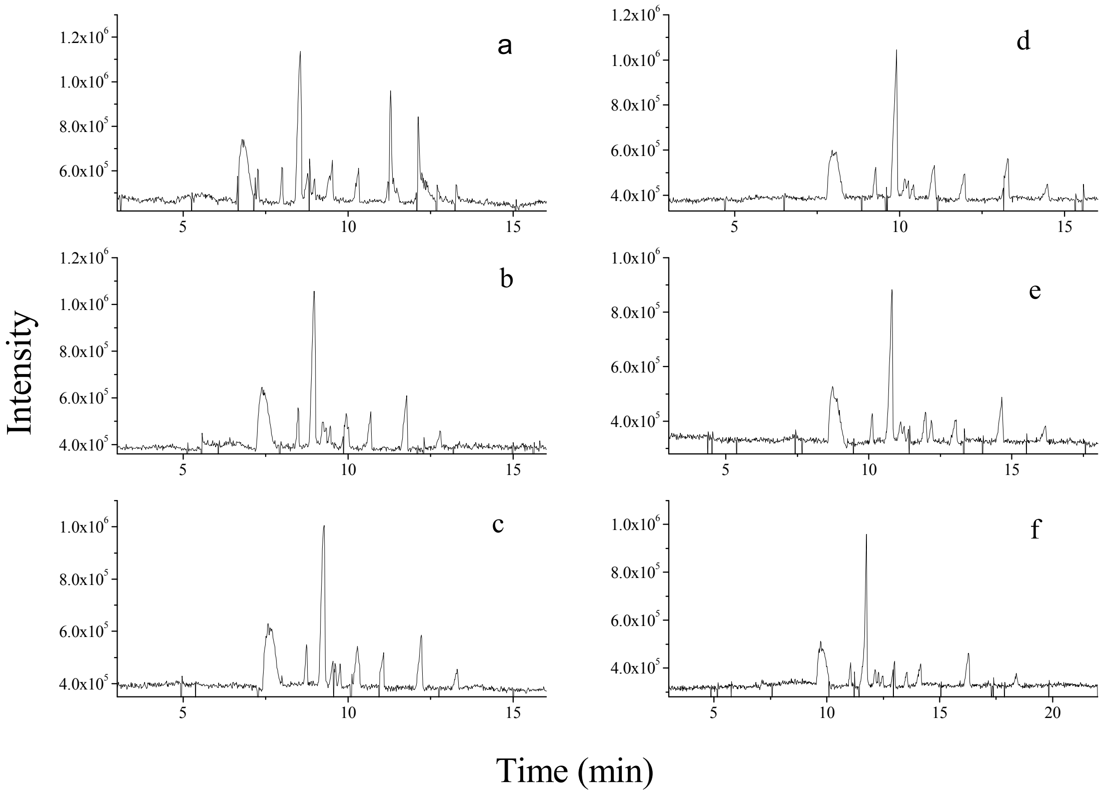

Figure S3. TIEs of mixed standards with different ratio of organic modifier (methanol) in buffer. (**a**) 0%, (**b**) 0.5%, (**c**) 1%, (**d**) 2%, (**e**) 5%, (**f**) 10%. The concentration of each diluted standard was 40 μg/mL.

Table S1. CE-ESI-TOF/MS accurate mass measurements of 15 nucleobase and nucleoside compounds.

Peak #	Compound ID	Formula	$[M+H]^+$	$[M+Na]^+$	$[2M+H]^+$	$[M+K]^+$	Product Ions
1	cytosine	$C_4H_5N_3O$	112.0507	-	223.0939	-	-
2	uracil	$C_4H_4N_2O_2$	113.0348	135.0171	-	151.0606	-
3	thymine	$C_5H_6N_2O_2$	127.0504	-	-	-	-
4	adenine	$C_5H_5N_5$	136.0618	-	-	-	-
5	hypoxanthine	$C_5H_4N_4O$	137.0459	159.0274	-	175.0018	-
6	guanine	$C_5H_5N_5O$	152.0569	174.0384	-	190.0123	-
7	xanthine	$C_5H_4N_4O_2$	153.0405	175.0230	-	-	-
8	thymidine	$C_{10}H_{14}N_2O_5$	243.0976	265.0799	-	281.0536	$[M-C_5H_8O_3+H]^+$ 127.0504
9	cytidine	$C_9H_{13}N_3O_5$	244.0933	266.0756	-	-	-
10	uridine	$C_9H_{12}N_2O_6$	245.0776	267.0589	-	283.0327	-
11	cordycepin	$C_{10}H_{13}N_5O_3$	252.1092	274.0914	-	-	-
12	adenosine	$C_{10}H_{13}N_5O_4$	268.1037	290.0860	-	-	-
13	2'-deoxyguanosine	$C_{10}H_{13}N_5O_4$	268.1037	290.0860	-	306.0598	$[M-C_5H_8O_3+H]^+$ 152.0567
14	inosine	$C_{10}H_{12}N_4O_5$	269.0881	291.0702	-	307.0436	-
15	guanosine	$C_{10}H_{13}N_5O_5$	284.0987	306.0753	-	322.0539	$[M+H-C_5H_8O_4]^+$ 152.0569

Note: Extracted ion electropherograms (EIEs) of 15 nucleobase and nucleoside standards are shown in Figure 1. Peak eluting earlier to peak 3 (in Figure 1) is a fragmentation ion of peak 8 (in Figure 1). The exact masses of peak 3 ($[M+H]^+$) and the fragmentation ion of peak 8 ($[M-C_5H_8O_3+H]^+$) were consistent with the same elemental composition of $C_5H_7N_2O_2$. Peak eluting after peak 9 (in Figure 1) is the C_{13} isotope peak of peak 8 ($[M+H]^+$). The exact masses of peak 9 ($[M+H]^+$) and the C_{13} isotope peak of peak 8 ($[M+H]^+$) were similar.
